# Multiple socioeconomic determinants of weight gain: the Helsinki Health Study

**DOI:** 10.1186/1471-2458-13-259

**Published:** 2013-03-22

**Authors:** Tina Loman, Tea Lallukka, Mikko Laaksonen, Ossi Rahkonen, Eero Lahelma

**Affiliations:** 1 Hjelt Institute, Department of Public Health, University of Helsinki, Helsinki, FIN-00014, Finland; 2 Finnish Institute of Occupational Health, Helsinki, Finland

**Keywords:** Socioeconomic position, Weight gain, Follow-up, Adulthood, Childhood, Cohort

## Abstract

**Background:**

Socioeconomic differences in weight gain have been found, but several socioeconomic determinants have not been simultaneously studied using a longitudinal design. The aim of this study was to examine multiple socioeconomic determinants of weight gain.

**Methods:**

Mail surveys were conducted in 2000–2002 among 40 to 60-year old employees of the City of Helsinki, Finland (n = 8 960, response rate 67%). A follow-up survey was conducted among the baseline respondents in 2007 with a mean follow-up of 5 to 7 years (n = 7 332, response rate 83%). The outcome measure was weight gain of 5 kg or more over the follow-up. Socioeconomic position was measured by parental education, childhood economic difficulties, own education, occupational class, household income, home ownership and current economic difficulties. Multivariable logistic regression models were fitted adjusting simultaneously for all covariates in the final model.

**Results:**

Of women 27% and of men 24% gained 5 kg or more in weight over the follow-up. Among women, after adjusting for age, baseline weight and all socioeconomic determinants, those with basic (OR 1.40 95% CI 1.11-1.76) or intermediate education (OR 1.43 95% CI 1.08-1.90), renters (OR 1.18 95% CI 1.03-1.36) and those with occasional (OR 1.19 95% CI 1.03-1.38) or frequent (OR 1.50 95% CI 1.26-1.79) economic difficulties had increased risk of weight gain. Among men, after full adjustment, having current frequent economic difficulties (OR 1.70 95% CI 1.15-2.49) remained associated with weight gain.

**Conclusions:**

Current economic difficulties among both women and men, and among women low education and renting, were associated with weight gain. Prevention of weight gain among ageing people would benefit from focusing in particular on those with economic difficulties.

## Background

According to the WHO guidelines, weight gain should not exceed five kilograms (kg) in adulthood [1,2]. However, weight gain is widespread and leads to health risks such as a higher risk of coronary heart disease [3] and type 2 diabetes [4]. Those in lower socioeconomic position are more likely to gain weight but the associations vary by socioeconomic determinants [5]. According to reviews, low parental socioeconomic position is associated with obesity in adulthood [6] and occupational class has shown the most consistent associations with weight gain [5]. For educational level and income the findings are less consistent, particularly among men.

Socioeconomic position covers a range of social, financial, non-material and material determinants from childhood to adulthood [7]. Socioeconomic position in general reflects one’s position in the socioeconomic hierarchy. The conventional determinants of socioeconomic position such as education, occupational class and income correlate with each other but are not interchangeable [7,8]. However, few studies have examined different socioeconomic determinants of weight gain simultaneously. According to a Swedish follow-up study, lower parental occupational class, educational level and occupational class were all associated with weight gain among women [9]. In a US cohort study, lower education, but not occupational class, was associated with greater weight gain [10]. Lower parental occupational class was associated with higher weight gain among women, and lower income among men. The associations remained after mutual adjustment for all socioeconomic determinants. In a British birth cohort study, body mass index (BMI) increased fastest among those with low parental occupational class, and the association remained after adjusting for education and occupational class [11]. Among middle-aged French men and women, lower education was associated with increased BMI but occupational class was not [12]. In a Dutch study, own occupational class was associated with weight gain among men and parental occupational class was associated with weight gain among women [13].

Our previous cross-sectional study showed that current economic difficulties and renting were associated with obesity among women, after adjusting for other indicators of socioeconomic position [14]. Among men, only economic difficulties in adulthood remained associated with obesity after full adjustment. In a longitudinal design the time sequence between the socioeconomic determinants and subsequent weight gain can be better established supporting causal interpretations of the associations. Previous longitudinal studies have examined only one or a couple of socioeconomic determinants at a time.

The aim of this study was to examine several socioeconomic determinants of weight gain among middle-aged women and men, and to examine the contribution of baseline weight and all socioeconomic determinants to the associations.

## Methods

### Data

In 2000, 2001 and 2002, baseline questionnaires were mailed to the employees of the City of Helsinki, Finland, who during the survey year reached the age of 40, 45, 50, or 60. The response rate was 67% (n = 8960). The proportion of women among the respondents was 80% which reflects the gender distribution among employees of the City of Helsinki. In 2007, follow-up questionnaires were sent to the baseline respondents. At follow-up, the response rate was 83% (n = 7 332). After excluding 25 pregnant women and respondents with missing data for socioeconomic determinants or weight (on average 1-2%), the final study population included 5 370 women and 1 252 men.

The Helsinki Health Study has received ethical approvals from the ethics committees of the Department of Public Health, University of Helsinki, as well as the City of Helsinki health authorities.

### Weight measures

Self-reported weight in kilograms was used to calculate weight change from baseline to follow-up. Weight gain of 5 kg or more was used as outcome following recommendations of the World Health Organization [1,2].

### Socioeconomic position

Seven determinants of socioeconomic position were included in the baseline survey. Parental education and childhood economic difficulties were included as indicators of childhood socioeconomic position. The educational level of both parents was asked, and the higher level was chosen to indicate parental education. Childhood educational level was classified into basic (primary school or less), intermediate (vocational school) and higher education (matriculation/college examination or university degree). Childhood economic difficulties were measured by asking whether there had been serious economic difficulties in the family before the respondent was sixteen years old.

The respondents’ own education and occupational class were used as indicators of adult socioeconomic position. Education was categorised into three levels: basic (primary school or less), intermediate (vocational school or matriculation/college examination) and higher (university degree). Due to changes in educational system, educational classifications are different between respondents and their parents, but they both reflect educational hierarchy. Data about occupational class were derived from the City of Helsinki personnel registers or completed from the questionnaires for those who did not consent to register linkages. Occupational class was classified into four hierarchical classes: professionals, semi-professionals, routine non-manual employees, and manual workers.

Indicators of current material resources were household income, home ownership and economic difficulties. The respondents were asked to report the overall net income of their household during a typical month. Income was weighted by the number of adults and children living in the household and categorised into quartiles, separately for women and men. Home ownership was categorised into owner-occupiers and renters. Economic difficulties were determined by whether or not respondents had enough money to buy food or clothing and whether they had difficulties in paying bills. The questions were: “How often do you not have enough money to buy the kind of food or clothing you or your family need?” and “How much difficulty do you have in meeting the payment of bills?”. Using the sum for the two questions, the responses were categorised into three groups: no, occasional or frequent economic difficulties. Further details of the measurement of socioeconomic determinants can be found in our earlier report [14].

### Statistical analysis

Mean baseline weight and weight change by gender and age were first computed. Second, age-adjusted prevalence of those who gained 5 kg in weight, by socioeconomic determinants, was computed using generalised linear models. Statistical significance was judged from 95% confidence intervals (95% CI). Third, logistic regression analysis was used to examine the associations between the seven socioeconomic determinants and the subsequent weight gain. Model 1 was adjusted for age, and model 2 additionally for baseline weight. Model 3 was fully adjusted for all covariates and socioeconomic determinants. Sensitivity analyses included also physical activity, binge drinking, smoking, vegetable consumption, mental health and reproductive health (data not shown). SPSS version 15.0 was used for the analyses.

## Results

Mean weight at baseline and weight change over the follow-up by gender and age are displayed in Table 1. Among women, mean weight was 68.5 kg [95% CI 68.2-68.9] at baseline and mean weight change 1.8 kg [95% CI 1.7-2.0]. Of women, 27% gained in weight 5 kg or more over the follow-up (Table 2). The prevalence of weight gain was lower among the most advantaged socioeconomic groups.

**Table 1:** Mean baseline body weight and weight change by age and gender

	**Women**	**Mean body weight at baseline (kg)**	**Weight change (kg)**
**Age**	n	kg [95% CI]	kg [95% CI]
40	1075	67.2 [66.4-67.9]	3.1 [2.7-3.4]
45	1150	67.6 [66.9-68.4]	3.1 [2.7-3.4]
50	1198	67.5 [66.8-68.2]	1.9 [1.6-2.3]
55	1330	70.1 [69.4-70.8]	1.0 [0.7-1.4]
60	617	70.5 [69.5-71.5]	0.0 [−0.5-0.4]
All	5370	68.5 [68.2-68.9]	1.8 [1.7-2.0]
	**Men**		
**Age**	n		
40	208	82.0 [80.1-83.8]	2.8 [2.1-3.6]
45	226	84.3 [82.5-86.0]	1.9 [1.2-2.7]
50	252	84.4 [82.8-86.1]	1.8 [1.1-2.5]
55	361	83.4 [82.0-84.8]	0.8 [0.2-1.4]
60	205	84.5 [82.6-86.3]	−0.8 [−1.6-(0.0)]
All	1252	83.7 [82.9-84.5]	1.3 [1.0-1.6]

**Table 2:** Age-adjusted prevalence (%) of weight gain (5 kg+) by age and socioeconomic determinants among women and men

	**Women**	**Weight gain ≥ 5 kg**	**Men**	**Weight gain ≥ 5 kg**
**Age**	n	% [95% CI]	n	% [95% CI]
40	1075	34 [31.7-36.9]	208	32 [26.1-37.4]
45	1150	34 [31.0-36.1]	226	33 [27.3-38.2]
50	1198	26 [23.3-28.3]	252	28 [23.0-33.3]
55	1330	21 [18.1-22.8]	361	18 [13.7-22.3]
60	617	14 [10.5-17.4]	205	10 [4.0-15.5]
**Parental education**				
Higher	1114	24 [21.3-26.5]	330	22 [17.6-26.7]
Intermediate	1416	27 [25.1-29.7]	313	25 [20.0-29.3]
Basic	2840	27 [25.4-28.6]	609	24 [20.6-27.3]
**Childhood economic difficulties**				
No difficulties	4403	26 [24.4-27.0]	1033	24 [21.3-26.4]
Difficulties	967	30 [27.2-32.7]	219	23 [17.2-28.2]
**Own education**				
Higher	1353	22 [19.5-24.1]	439	22 [17.7-25.5]
Intermediate	2969	28 [26.2-29.4]	584	25 [21.8-28.6]
Basic	1048	29 [26.1-31.4]	229	24 [18.0-28.9]
**Occupational class**				
Professionals	1474	24 [21.4-25.8]	571	21 [17.9-24.7]
Semi-professionals	1090	25 [22.7-27.9]	255	24 [18.5-28.8]
Routine non-manual employees	2110	29 [27.1-30.8]	124	26 [19.0-33.8]
Manual workers	696	27 [23.8-30.3]	302	27 [22.2-31.6]
**Household income**				
Highest quartile	1298	26 [23.3-28.1]	314	20 [15.4-24.8]
2nd	1104	23 [20.8-26.0]	307	22 [17.5-26.8]
3rd	1521	28 [25.3-29.6]	316	24 [18.9-28.1]
Lowest quartile	1447	29 [26.2-30.7]	315	29 [24.1-33.4]
**Home ownership**				
Owner-occupiers	3593	24 [23.0-25.8]	888	22 [18.9-24.4]
Renters	1777	31 [28.6-32.7]	364	29 [24.2-32.8]
**Economic difficulties**				
No difficulties	2821	23 [21.6-24.8]	694	20 [17.0-23.3]
Occasional difficulties	1585	28 [25.8-30.1]	359	25 [21.0-29.7]
Frequent difficulties	964	34 [30.8-36.3]	199	33 [27.0-38.5]
**Total**	5370	27 [25.3-27.7]	1252	24 [21.2-26.0]

Among men, mean weight was 83.7 kg [95% CI 82.9-84.5] at baseline and mean weight change 1.3 kg [95% CI 1.0-1.6] (Table 1). Of men, 24% gained in weight 5 kg or more over the follow-up (Table 2). The prevalence of weight gain was higher among those who had frequent current economic difficulties than among those who did not have such difficulties.

Adjusting for age, each socioeconomic determinant except household income was associated with weight gain among women (Table 3). For the socioeconomic determinants, the most disadvantaged group had a higher likelihood of gaining 5 kg or more (Model 1). Those with frequent economic difficulties (OR 1.68 95% CI 1.43-1.98) and basic education (OR 1.48 95% CI 1.22-1.80) were most likely to gain weight. The effect of adjusting for baseline weight (Model 2) was small. However, the associations between childhood socioeconomic position, parental education and childhood economic difficulties and weight gain, lost statistical significance when baseline weight was adjusted for. After full adjustment for age, baseline weight and all socioeconomic determinants, those with basic (OR 1.43 95% CI 1.08-1.90) or intermediate (OR 1.40 95% CI 1.11-1.76) education, renters (OR 1.18 95% CI 1.03-1.36) and those with occasional (OR 1.19 95% CI 1.03-1.38) or frequent (OR 1.50 95% CI 1.26-1.79) economic difficulties in adulthood were more likely to gain weight (Model 3).

**Table 3:** **Associations between socioeconomic determinants and weight gain (5 kg+), odds ratios (OR) and their confidence intervals (95% CI**) **among women (n = 5391)**

**Weight gain ≥ 5 kg**	**Model 1**	**Model 2**	**Model 3**
	**OR [95% CI]**	**OR [95% CI]**	**OR [95% CI]**
**Parental education**			
Higher	1.00	1.00	1.00
Intermediate	1.20 [1.01-1.44]	1.17 [0.97-1.40]	1.06 [0.88-1.28]
Basic	1.19 [1.01-1.40]	1.15 [0.97-1.35]	1.01 [0.84-1.20]
**Childhood economic difficulties**			
No difficulties	1.00	1.00	1.00
Difficulties	1.19 [1.02-1.39]	1.16 [1.00-1.35]	1.12 [0.95-1.32]
**Own education**			
Higher	1.00	1.00	1.00
Intermediate	1.39 [1.19-1.62]	1.33 [1.14-1.55]	1.40 [1.11-1.76]
Basic	1.48 [1.22-1.80]	1.38 [1.14-1.68]	1.43 [1.08-1.90]
**Occupational class**			
Professionals	1.00	1.00	1.00
Semi-professionals	1.14 [0.96-1.37]	1.12 [0.93-1.34]	0.84 [0.66-1.06]
Routine non-manual employees	1.34 [1.15-1.56]	1.28 [1.10-1.49]	0.92 [0.72-1.17]
Manual workers	1.26 [1.03-1.55]	1.18 [0.96-1.44]	0.77 [0.57-1.04]
**Household income**			
Highest quartile	1.00	1.00	1.00
2nd	0.88 [0.73-1.07]	0.88 [0.73-1.07]	0.81 [0.67-0.99]
3rd	1.10 [0.92-1.30]	1.09 [0.92-1.29]	0.94 [0.78-1.13]
Lowest quartile	1.15 [0.96-1.37]	1.14 [0.96-1.35]	0.87 [0.72-1.06]
**Home ownership**			
Owner-occupiers	1.00	1.00	1.00
Renters	1.39 [1.23-1.58]	1.33 [1.17-1.51]	1.18 [1.03-1.36]
**Economic difficulties**			
No difficulties	1.00	1.00	1.00
Occasional difficulties	1.29 [1.12-1.49]	1.23 [1.07-1.42]	1.19 [1.03-1.38]
Frequent difficulties	1.68 [1.43-1.98]	1.60 [1.37-1.87]	1.50 [1.26-1.79]

**Model 1** Adjusted for age.

**Model 2** Model 1 and baseline weight adjusted for.

**Model 3** Model 2 and all socioeconomic indicators adjusted for.

Adjusting for age, household income, home ownership and current economic difficulties were associated with weight gain among men (Table 4). Those with the lowest household income (OR 1.64 95% CI 1.11-2.42), renters (OR 1.40 95% CI 1.06-1.84) and those with frequent economic difficulties in adulthood (OR 1.96 95% CI 1.37-2.80) were more likely to gain 5 kg or more (Model 1). As for women, adjusting for baseline weight made mostly minor contributions to the associations examined (Model 2). Only economic difficulties remained associated with weight gain (OR 1.70 95% CI 1.15-2.49) after full adjustment (Model 3).

**Table 4:** Associations between socioeconomic determinants and weight gain (5 kg+), odds ratios (OR) and their confidence intervals (95% CI) among men (n = 1247)

**Weight gain ≥ 5 kg**	**Model 1**	**Model 2**	**Model 3**
	**OR [95% CI]**	**OR [95% CI]**	**OR [95% CI]**
**Parental education**			
Higher	1.00	1.00	1.00
Intermediate	1.16 [0.80-1.67]	1.14 [0.79-1.65]	1.13 [0.77-1.65]
Basic	1.11 [0.80-1.54]	1.09 [0.78-1.51]	1.09 [0.76-1.56]
**Childhood economic difficulties**			
No difficulties	1.00	1.00	1.00
Difficulties	0.99 [0.70-1.39]	0.99 [0.70-1.39]	0.81 [0.56-1.17]
**Own education**			
Higher	1.00	1.00	1.00
Intermediate	1.23 [0.91-1.66]	1.14 [0.84-1.55]	0.88 [0.58-1.31]
Basic	1.12 [0.75-1.67]	1.03 [0.69-1.54]	0.66 [0.38-1.16]
**Occupational class**			
Professionals	1.00	1.00	1.00
Semi-professionals	1.12 [0.78-1.60]	1.07 [0.75-1.54]	1.10 [0.71-1.70]
Routine non-manual employees	1.34 [0.87-2.07]	1.28 [0.82-1.99]	1.11 [0.64-1.92]
Manual workers	1.37 [0.99-1.87]	1.30 [0.94-1.80]	1.31 [0.82-2.10]
**Household income**			
Highest quartile	1.00	1.00	1.00
2nd	1.16 [0.77-1.74]	1.05 [0.71-1.56]	1.11 [0.73-1.67]
3rd	1.25 [0.84-1.87]	1.14 [0.77-1.68]	1.13 [0.74-1.72]
Lowest quartile	1.64 [1.11-2.42]	1.48 [1.02-2.16]	1.35 [0.88-2.08]
**Home ownership**			
Owner-occupiers	1.00	1.00	1.00
Renters	1.40 [1.06-1.84]	1.34 [1.02-1.78]	1.26 [0.92-1.71]
**Economic difficulties**			
No difficulties	1.00	1.00	1.00
Occasional difficulties	1.37 [1.00-1.86]	1.28 [0.95-1.73]	1.25 [0.91-1.72]
Frequent difficulties	1.96 [1.37-2.80]	1.68 [1.18-2.38]	1.70 [1.15-2.49]

**Model 1** Adjusted for age.

**Model 2** Model 1 and baseline weight adjusted for.

**Model 3** Model 2 and all socioeconomic indicators adjusted for.

## Discussion

### Main findings

This study examined the associations of multiple socioeconomic determinants with gaining 5 kg or more among middle-aged women and men. After full adjustment, women with lower education, renters and those who had economic difficulties in adulthood were more likely to gain weight than their higher position counterparts. Among men, only frequent economic difficulties in adulthood remained associated with weight gain after baseline weight and all other socioeconomic determinants had been taken into account.

### Interpretation

Conventional socioeconomic determinants had inconsistent and weak associations with weight gain. This finding is in accordance with previous studies [5,10,13]. There was an association between education and weight gain among women, and this association remained after full adjustment. After full adjustments, most of the other associations between socioeconomic determinants and weight gain disappeared. This indicates that there are interrelationships between the various determinants of socioeconomic position. For example, education precedes occupation and explains part of the association between occupational class and weight gain.

Socioeconomic differences in weight may occur even before adulthood [15], and early adulthood is potentially important to the prevention of weight gain [16]. Among the middle-aged prevention of weight gain needs to focus in particular on those in low socioeconomic positions. Previous studies have found an association between parental socioeconomic position and adult weight gain [11,13]. However, this was not found in our cohort. This discrepancy may be based on different study designs, as the previous studies have followed their cohorts from childhood on, while our data on parental position were based on retrospective reports.

It is not well understood why weight gain and its social, cultural and environmental determinants differ by socioeconomic position [5]. For example, marital status, food habits, and physical activity have been associated with increased body weight, but in an earlier study [17] these variables failed to explain the socioeconomic differences in weight. In a middle-aged cohort from the UK, occupational class remained associated with weight gain even after adjustment for baseline BMI, smoking, physical activity and diet [18].

Imbalances in energy intake and expenditure can accumulate over time and contribute to weight gain. Socioeconomic position may influence behaviours such as physical activity [5]. Thus, associations between socioeconomic position and weight may be partly mediated through physical inactivity and diet [19]. Furthermore, economic and cultural circumstances in society may particularly favour weight control behaviours among those in higher socioeconomic positions [20]. In our earlier study, those with higher occupational class were more physically active [21], and their food habits better followed the national guidelines [22]. This can contribute to socioeconomic differences in weight gain. Among men only, taking into account physical inactivity only slightly attenuated the association between current economic difficulties and weight gain. Our sensitivity analyses also included daily fresh vegetable consumption, binge drinking, smoking and reproductive health but they had negligible effects on the studied associations (data not shown).

In our study, childhood socioeconomic determinants were unassociated with weight gain after mutual adjustments. In previous studies, associations have been more consistent among women than among men, but these studies have used parental occupational class instead of education [11,13]. In our study, childhood socioeconomic position was not associated with weight gain after taking into account current socioeconomic position. Childhood circumstances may no longer have effects on weight gain in late middle-age. In a US study, parental socioeconomic position was associated with weight gain only among younger adults, after taking into account current socioeconomic position [10]. Current socioeconomic circumstances in adulthood may be more relevant to weight gain than parental socioeconomic position.

Women and men who had economic difficulties in adulthood were more likely to gain 5 kg in weight. Economic difficulties in adulthood and renting were associated with obesity among women in our previous cross-sectional study [14]. In the present study, household income did not increase the likelihood of gaining weight, but current economic difficulties did. Among US men, job insecurity, instability and decrease in income have been associated with weight gain [23]. Among women, material resources such as being an owner-occupier were a protective factor against weight gain in the present study. In stressful life situations caused by economic difficulties, people may have less time and energy to maintain a stable weight [24]. Common mental disorders have also been associated with economic difficulties [25]. However, in our study common mental disorders did not attenuate the associations between socioeconomic determinants and subsequent weight gain (data not shown).

### Methodological considerations

There were some limitations in the present study. We used self-reported weight which is likely to be underreported. Self-reported and measured weights are, however, strongly correlated [16]. A recent study showed that self-reported BMI can be used as a valid predictor of a health-related outcome [26]. Over a 5–7 year follow-up period, weight gain of 5 kg is a major change, and the WHO recommends that weight gain should not exceed 5 kg during adult life [1,2]. To confirm that the results are not sensitive to the chosen criteria, we also conducted sensitivity analyses using 5% weight gain as an outcome and these analyses produced similar results (data not shown).

The response rates to our surveys were acceptable at baseline (67%) and at follow-up (83%), but non-participation is still a challenge and a potential source of bias. Non-response analyses have shown that manual workers, men, younger employees, and those with long sickness absence spells were only slightly overrepresented among the non-respondents, and the data largely represents the target population [27,28]. The small and partly inconsistent differences between the participants and non-participants are unlikely to cause major bias to the associations studied. Childhood determinants have been collected retrospectively and may include recall bias.

All respondents were from the Helsinki metropolitan area and were employed at baseline by the City of Helsinki. Thus, the results cannot be generalised to the general population of Finland, and not even to the employed population at large.

## Conclusions

Considering a broad range of socioeconomic determinants, economic difficulties in adulthood remained associated with weight gain among both women and men. Prevention of weight gain among ageing people would benefit from focusing in particular on those with economic difficulties.

## Competing interests

The authors declare that they have no competing interests.

## Authors’ contributions

Each author has contributed to the planning of the study and analysis, commented and revised the manuscript text, as well as approved submission of the final version. TLo conducted the analyses and drafted the first version of the manuscript.

## Pre-publication history

The pre-publication history for this paper can be accessed here:

http://www.biomedcentral.com/1471-2458/13/259/prepub
